# Effectiveness of an Educational Intervention of Breast Cancer Screening Practices Uptake, Knowledge, and Beliefs among Yemeni Female School Teachers in Klang Valley, Malaysia: A Study Protocol for a Cluster-Randomized Controlled Trial

**DOI:** 10.3390/ijerph17041167

**Published:** 2020-02-12

**Authors:** Sarah Noman, Hayati Kadir Shahar, Hejar Abdul Rahman, Suriani Ismail

**Affiliations:** Department of Community Health, Faculty of Medicine & Health Sciences, Universiti Putra Malaysia, Serdang 43400, Malaysia; saranoman12@gmail.com (S.N.); hejar@upm.edu.my (H.A.R.); si_suriani@upm.edu.my (S.I.)

**Keywords:** breast cancer, breast cancer screening, knowledge, beliefs, educational intervention, Yemeni teachers

## Abstract

Breast cancer is the most common cancer spread among women worldwide. Whereas many studies have discussed the significance of breast cancer screening among women in various countries, few have attempted to discuss this topic among female school teachers. As teachers educate and communicate with students, this may play an essential role in health education and in promoting healthy behavior, such as breast cancer screening. The primary goal of this study is to develop and implement an educational intervention of breast cancer screening and evaluate its effectiveness among Yemeni female school teachers in Malaysia. This was carried out as per the health belief model. A cluster-randomized controlled trial was conducted among 183 Yemeni female school teachers in twelve schools in Klang Valley, Malaysia. A random assignment of the target schools was made to include them within the intervention or control group. Participants in the intervention group were offered a 90-min session for one-day educational intervention on breast cancer screening. On the other hand, participants in the control group were offered the same educational materials at the end of the study. Relevant data was collected at baseline, one month following the intervention, and then three- and six-months follow-up assessments. Analysis of such data was done via IBM SPSS software 25.0 by generalized estimating equations (GEE) to assess the differential changes over time. A primary outcome embodied in breast cancer screening practice uptake was expected. Secondary outcomes include the target group’s knowledge on and beliefs of breast cancer screening. This study intends to contribute to the credibility and effectiveness of utilizing a theory-based breast cancer screening intervention in order to raise the awareness of women on conducting breast cancer screening.

## 1. Introduction

Although efforts and extensive progress have been made in medical treatment, breast cancer (BC) continues to be among the highest life-threatening diseases among women [1]. It is argued that BC mortality rates in many countries with low and middle income are higher than those in high-income countries. This could be attributed to the early diagnosis of BC in the latter countries. Unfortunately, the diagnosis of BC in the former countries is made at later stages [2]. Yemen, as one of the low-income countries, is a case in point. Most BC cases are identified at late stages or after the occurrence of metastasis [3]. Due to the recent instability of Yemen and lack of security, many Yemenis decided to immigrate to Malaysia for educational purposes and/or job opportunities or to reside as immigrants. As a result, Yemenis in Malaysia have reached a population of about 27,000 according to 2018 estimates of the Yemeni Embassy. Among these are Yemeni females who may not have any idea of BCS services and lack the knowledge and good health beliefs in this regard.

Practically speaking, BC early detection examinations help save thousands of lives each year and improve the chances for early detection of BC, and therefore successful treatment [4]. Several approaches have been evaluated in this respect, such as BCS methods, including BSE, clinical breast exam (CBE), and mammography [5]. A number of epidemiological studies have been conducted on community samples of various women groups. Such studies have shown that the rate of BCS practice was low in various countries [6,7,8]. In Yemen, the findings of similar studies on BSE show that the rate of women performing BSE ranges from 11% to 17.4% [9,10]. 

Theoretically, different models and theories have been used in order to comprehend BC early detection. One is the health belief model (HBM) that is used as the theoretical framework in numerous educational interventions. HBM has proven to be effective in improving factors impacting BCS behaviors [7,11,12,13,14]. The theoretical framework of the HBM emphasizes that threats pertaining to health concerns can negatively affect the health behavior of women. For instance, women with the feeling of being susceptible to BC risk are more likely to do BSE. Women, having good health motivation, gaining higher benefits and having fewer barriers to a breast exam are more probable to do BSE [15]. Moreover, knowledge about BCS practice is associated with an increase in performing BCS behaviors, as suggested by the model [16]. 

Many studies on BCS performance have been implemented among women worldwide. Such studies have incorporated certain women groups such as female university students, female workers, women attending health care centers, or female residents [12,17,18,19,20]. Nevertheless, only a few studies have been conducted on female teachers regarding BCS practice in the world [11,21,22,23,24,25]. For example, in Malaysia, even though a few studies have shed light on BCS practice among teachers, no research has been done on BCS practice among Yemeni female school teachers in Malaysia. As teachers, they are regarded as role models and an essential educational source. They could possibly play a vital role in offering health education and in promoting healthy behavior pertaining to BCS practice to be adopted by future generations. In addition, one day, these teachers will return to their home country and, thus, spread the knowledge they have gained in this domain. Therefore, developing and implementing this BC educational program among Yemeni female school teachers could be one of the effective means to reduce BC mortality rates within this group and develop educational programs for other groups.

In brief, interventions focusing on BC early detection are less common among school teachers, in general, and absent among Yemeni school teachers in Malaysia, in particular. Therefore, not only does the current study intend to develop and evaluate an educational intervention based on the HBM but also to educate Yemeni female school teachers in Arabic schools in Malaysia and provide them with knowledge and enhance their beliefs of BC and BCS practice. It also aims to promote BCS among them.

The overall objective of this study is to develop and evaluate the effectiveness of an educational intervention grounded in the HBM on BCS uptake (BSE, CBE, and mammography), knowledge, and beliefs among Yemeni female school teachers in Klang Valley, Malaysia. It is hypothesized that starting from baseline until the three- and six-month assessment, women in the experimental group will gain the following:

i. An increase in the proportion of their BCS uptake
ii. An increase in their BC knowledge scores
iii. An improvement in their BC health beliefs (perceived susceptibility, perceived seriousness, perceived benefits, perceived barriers, health motivation, confidence).

## 2. Materials and Methods

### 2.1. Study Design

This is a parallel cluster-randomized controlled trial (cRCT), with schools being the unit of randomization (clusters). The trial will be used to assess the effectiveness of an educational intervention on BCS uptake, knowledge, and beliefs of Yemeni female school teachers at both elementary and high Arabic schools in Klang Valley, Malaysia. There are nineteen well-known Arabic schools in Klang Valley area, with teachers from different Arab countries (i.e., Yemen, Iraq, Egypt, Libya, Saudi Arabia). However, the main composition of the teaching bodies is Yemeni teachers, as six of the schools are Yemeni schools. Twelve of the schools were selected as clusters (schools), and randomly assigned for the intervention or the control group. While the intervention group was given an educational intervention on BCS, the control group received the educational materials on BCS intervention after the completion of the study. 

### 2.2. Eligibility Criteria

The inclusion criteria for schools will take into account the following requirements: (1) such samples are elementary and high Arabic schools; (2) they are situated in Klang Valley; (3); and they consent to participate in the study. The exclusion criteria will disqualify Arabic schools that do not have Yemeni female teachers from the study,

The inclusion criteria for teachers takes into consideration the following requirements: (1) such participants must only be Yemeni female school teachers teaching at the selected elementary and high Arabic schools situated in Klang Valley; (2) participants are aged 20 years old or older; (3) they sign a consent form to take part in the study. The exclusion criteria for teachers include (1) teachers who are retiring during the study, (2) teachers who have been diagnosed with BC, and (3) teachers who are lactating or pregnant.

### 2.3. Intervention 

The intervention group will be introduced to an educational intervention on BCS. This intervention is grounded in the HBM and developed based on the American Cancer Society [4], American Congress of Obstetricians and Gynecologists [26], and the International Agency for Research on Cancer (IARC) [27]. Many educational interventions, being grounded in the HBM, have shown improvement in the practice of BCS behaviors on the part of relevant participants. The educational intervention module uses six constructs of the HBM: perceived susceptibility, perceived seriousness, perceived benefits, perceived barriers, health motivation, confidence. This intervention was prepared and designed to bridge the gap of BC knowledge and to modify beliefs related to BC. Table 1 gives an outline of the educational intervention on BCS along with the application of the HBM concepts in the educational intervention. 

The educational intervention consists of four units. Unit One provides general information on the anatomy and physiology of a normal breast in order for the participants to have a clear understanding of the topic. Unit Two gives information and knowledge of BC. It further explores BC symptoms, BC stages, BC risk factors in order to increase the participants’ knowledge of BC. Unit Three offers an explanation of two different methods of BCS (CBE and mammography) in a bid to encourage participants to adopt and practice these approaches. Unit Four explains the BSE procedure to raise the participants’ awareness of BC symptoms and motivate them to follow this procedure. 

Following the development of the educational intervention, face and content validity were checked and assessed by five professional expert panelists from the Community Health Department and Cancer Resource and Education Centre at the Faculty of Medicine and Health Sciences, Universiti Putra Malaysia. The experts approved the educational material as efficient with respect to the study objectives. Their feedback focused on the understandability and simplicity of the content. They suggested that information about the incidence of BC and any medical terms from the module ought to be removed because it is irrelevant to the layperson, a glossary of terms for the module ought to be drafted, and colorful pictures for the module should be included in order for the module to be clearer and more interesting. 

The educational intervention involves a 90-min session for one-day. The study researcher with the help of a trained research assistant, will conduct the session for the intervention group at each respective school. It is expected that three sessions will be carried out each week. Although the implementation date of the intervention may differ from one school to another, its duration time will be the same for all. The following is a breakdown of the procedures to be conducted:

(a) A 60-min PowerPoint presentation will be delivered along with a revised five-minute short video about BSE performance provided by the permission of Nuffield Health [28]. Another five-minute short video will be shown, highlighting the testimony made by one of the BC survivors. In other words, the BC survivor will share her experience with BC and give some advice on the importance of BCS and early detection.
(b) A thirty-minute training session on BSE practice on a silicone breast model with several implanted lumps will be carried out. In this session, participants will acquire knowledge on palpation technique and search strategy as well as signs of BC in order to be aware of when examining the breast. Following that, participants will be asked to perform BSE based on what they have learnt.
(c) At the end of the educational intervention, participants will be offered a copy of the relevant booklet containing all the information delivered to them in the educational intervention; they will be given CDs of the five-minute short video about BSE performance, and a BC logo sticker to be hung on the participants’ mirrors. The significance of distributing such materials lies in reminding the participants of the high importance of BCS practice. Along with encouraging the performance of BSE on a monthly basis, this aims at enhancing the intended messages.
(d) All participants in the intervention group will continue to receive short reminder text messages for a period of 6 months. The text message will read as: “This is a gentle reminder of BSE monthly performance and BCS practice.” The key messages will provide recommendations about BCS behaviors to remind, motivate, and encourage participants to practice BCS behaviors.

Furthermore, participants in the control group will not receive any education during the study period. However, they will be given the same educational materials on BCS at the end of the study, and they also will answer the same sets of the questionnaires at baseline, one month following the intervention, then after three- and six-months have gone by after the intervention. 

#### Intervention Fidelity

To support the fidelity of the educational intervention, some strategies are used. These include the strategy for preserving the consistency of the intervention delivery and for encouraging participants’ adherence to the intervention and reinforcing such abidance on their part. To maintain consistency in the intervention delivery, the educational intervention is implemented independently in each study cluster by the same researcher. The researcher will be committed to adopt a single educational intervention protocol to ensure a standard delivery across the respective schools. 

BSE training will be planned and rehearsed by the researcher by using a role-play technique with the participants. The researcher acts as an observer during the sessions and will provide immediate feedback to the participants. Other possible means enhancing intervention fidelity include the monthly short reminder text messages and the use of the BC logo sticker. These are mainly employed to encourage participants, remind them of the importance of BCS practice and monthly BSE performance, and to reinforce the intended messages.

### 2.4. Participant Privacy

To ensure sincere responses, honesty will be emphasized to the participants. They will be informed that all questionnaires will be anonymous with a unique number to identify participants later on. 

### 2.5. Outcome Measures

#### 2.5.1. Primary Outcomes

The primary outcome variables in the present study include

##### The Proportion of Participants Who Perform BSE Regularly and Proficiently

BSE practice and performance frequency will be measured using a self-report answer instrument. Developed by Akhtari-Zavare et al. [12], this instrument incorporates the following questions: (a) Have you ever performed BSE [yes/no], (b) How often do you perform BSE (once a month, once in 2–3 months, other, never). 

A woman who does BSE once a month will be classified as practicing (regular BSE); any other answer will be classified as non-practicing (irregular BSE). The proficiency of performing BSE will be measured by asking the participant to perform BSE on a breast model through BSE proficiency rating instrument (BSEPRI) [29]. This instrument is a checklist assessing the participants’ skills of BSE performance. This assessment is done while the participants demonstrate BSE procedures on a breast model. Needless to say, higher scores indicate greater BSE performance.

##### The Proportion of Participants Who Attend a Clinic for Screening CBE and Mammography

Screening practice regarding CBE and mammography will be assessed using self-report questions assessing participants as “practicing” or “not practicing”.

Using self-reporting in some of the primary outcomes may lead to some sort of bias judgment. Although unavoidable, the researcher tries to reduce this prejudice by being more objective at the data collection stage. This is by using simply precise language, maintaining a short time frame, and assuring confidentiality and anonymity of participants.

#### 2.5.2. Secondary Outcomes

The secondary outcome variables in this study include

##### Knowledge of Breast Cancer

Knowledge of BC and BCS methods will be measured using a modified questionnaire consisting of items adapted from Parsa [30], Akhtari-Zavare et al. [12], McCance et al. [31]. This self-administrated questionnaire consists of 35 items; these include symptoms of BC (6 items), BC risk factors (14 items), breast health awareness (5 items), and BCS methods (10 items). Responses will be evaluated by using the options of the (true, false, I do not know) nominal scale. One score or point will be given for every right answer while zero points for every wrong or uncertain response. The total reliability score for knowledge of BC questionnaire for this study has an acceptable reliability coefficient (α = 0.89). 

##### Beast Cancer Health Beliefs

Health beliefs pertaining to BC will be assessed by the use of a modified version of the CHBMS Champion [32]. The questionnaire consists of 63 self-report measures scales on ten subscales. These are represented as follows: perceived susceptibility to BC (5 items), perceived seriousness of BC (7 items), perceived benefits of BSE (6 items), perceived barriers for BSE (6 items), confidence in one’s ability to do BSE (11 items), health motivation (7 items), benefits of CBE (4 items), barriers of CBE (6 items), benefits of mammography (6 items), and barriers of mammography (5 items). All items will be measured by using the five-point Likert scale via the following list of options: strongly disagree (1 point), disagree (2 points), neutral (3 points), agree (4 points), and strongly agree (5 points). Scores will then be calculated or summated for analysis. With the exception of the barrier item, all other scales are positively related to screening behaviors. The total score reliability for the HBM scale for this study has an acceptable reliability coefficient (α = 0.85).

#### 2.5.3. Other Outcomes

##### Participants’ Personal Information

This part of the questionnaire consists of five questions conducive for assessing the socio-demographic factors of the participants (i.e., age, marital status, education level, income, family history of cancer). In addition, there is another question on previous reading or hearing about BCS.

Lawshe’s method [33] was used to examine the content validity index (CVI) of the questionnaire, indicating a good CVI (CVI = 0.95%). Moreover, the face validity of the questionnaire was initially scrutinized by five professional expert panelists from the Community Health Department and Cancer Resource and Education Centre at the Faculty of Medicine and Health Sciences, Universiti Putra Malaysia. Then some changes on the relevant items were made depending on the suggestions provided by these professional experts. 

### 2.6. Translation of the Questionnaire and Study Module

The questionnaire and study module were translated according to the process of professional translation, while the back translation was used as an approach for the adaptation of instruments [34]. The administration of forward-translation of the original module and questionnaires from English to Arabic language was carried out by an independent PhD health professional from the University of Malaya. The health professional is a native speaker of Arabic and an expert in English and familiar with the terminology of the study topic. With the assistance of the original translator, two bilingual PhD students in English and Arabic languages from Universiti Putra Malaysia were asked to review the translated module and questionnaire. Further, an independent PhD student translator from Universiti Putra Malaysia, whose mother tongue is English and who does not have any knowledge of the module and questionnaire, translated the module and questionnaire back to English. Those translators were instructed to construe the content and confirm the conceptual meanings with an emphasis on the use of acceptable and natural language. Translated versions of the module and questionnaire were compared with the original versions by the study researcher, who then decided the accuracy of the final version of the module and questionnaire. 

Later on, the translated module and questionnaire were pre-tested among 30 Yemeni female school teachers, who are not of the participants in the study. The clarity, understandability, and quality of the module and questionnaire were discussed with them individually. Then, phrases that were unsuitable and/ or difficult to understand were identified, and accordingly, adjustments and modifications thereto were made following their evaluation. Subsequently, the final version was approved for the study.

### 2.7. Sample Size

For the sake of calculating the appropriate sample size for clustering, the standard sample size estimate for the individually randomized design will be calculated. This calculation process is done first by applying the formula to find the difference between two population proportions (power = 0.80, alpha = 0.05 two-sided), (P1 = 0.71, P2 = 0.43 [35]). Then it is inflated by the design effect (design effect = 1.4). The power calculations propose a minimum sample size of 47 participants per arm. This is done by taking into account 20% attrition and 10% expected proportion eligibility. Therefore, a minimum of 183 participants is required for this study.

(1) $$N\;=\;{\left\{1.96\surd 1.14*0.43\;+\;0.84\surd 0.21\;+\;0.25\right\}}^{2}/{\left(0.71\;-\;0.43\right)}^{2}\;=\;47$$

### 2.8. Sampling Method

A total number of 12 schools with 183 participants that have met the inclusion and exclusion criteria will take part in the study. Following cluster sampling, six of the schools will be involved in the intervention group, while the other six are the control group. Then the proportionate allocation number of teachers to participate in the study will be calculated based on the density of the teachers in each school (probability proportional to size (PPS). Participants’ selection will be conducted by using a simple random sampling technique.

### 2.9. Participant Recruitment 

Nineteen Arabic schools with 289 Yemeni teachers were assessed for eligibility. Seven schools with 39 teachers were not eligible to take part in the study. In other words, whereas three schools did not show any will and interest to take part in the study, the remaining four schools did not meet the inclusion criteria as they did not include any Yemeni teachers.

With respect to the 12 eligible schools (12 schools), they will be invited to contribute to the study. Written consent will be obtained from respective authorities in all the schools. With the help of school coordinators, eligible teachers would be invited to attend small meeting groups in each school. During such meetings, the researcher will explain the objectives and advantages of the study, along with the inclusion and exclusion criteria. After selecting the required participants’ number in each school, participants will be asked for written informed consent. Accordingly, participants who agree to participate will be asked to fill in the questionnaire. Later on, educational interventions on BCS will be delivered to the intervention group. Following that, the participants will be requested to fill in the same set of the questionnaires (with the exception of the personal information form) in the post and follow-up assessment. 

### 2.10. Study Principles

The reporting of this protocol followed the Standard Protocol Items: Recommendations for Interventional Trials (SPIRIT) 2013 Statement [36]. The study reporting will be in accordance with the Consolidated Standards of Reporting Trials (CONSORT) statement.

**Figure 1:**
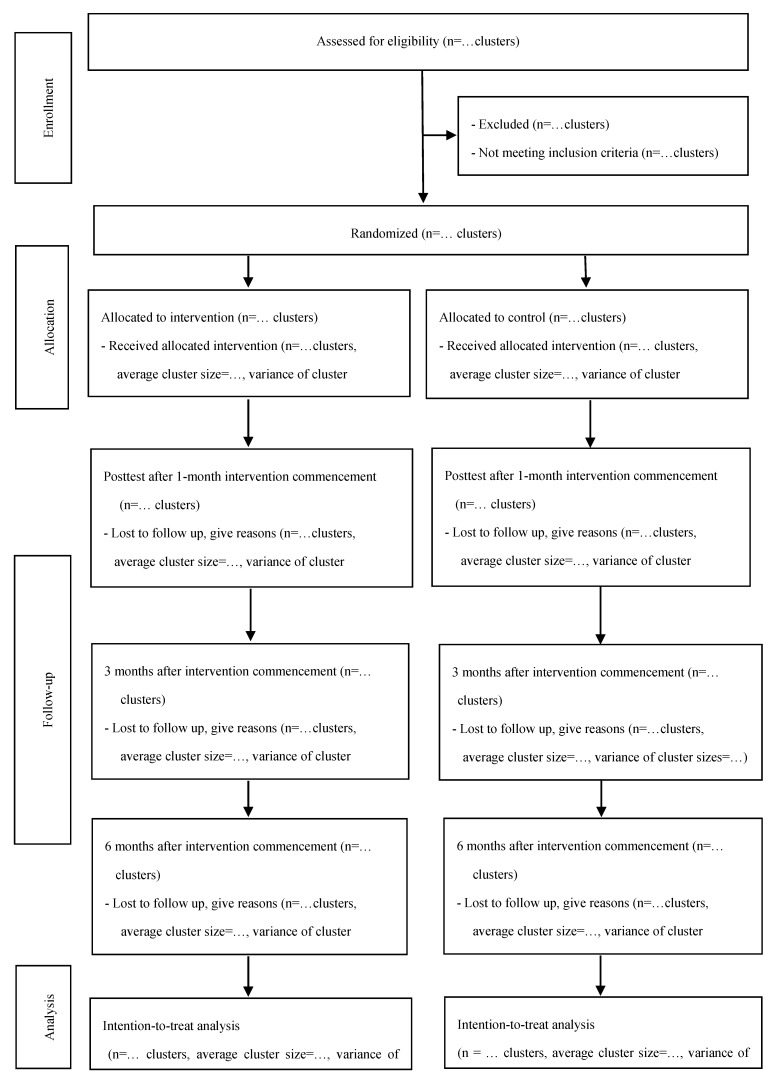
CONSORT flow diagram of the study—adapted from Campbell et al. [37].

Figure 1 below illustrates the Consolidated Standards of Reporting Trials (CONSORT) flowchart [37].

### 2.11. Randomization

The unit of randomization in this current study is a school. To allocate the twelve eligible clusters (schools) to the study groups, an independent statistician will create the randomization sequence using the Sealed Envelope tool [38], with a 1:1 allocation using random block sizes of 2 and 4. Thus, six clusters were randomly allocated to the intervention group (VX9, XP6, EP9, ZI8, FF5, and IQ3) and six clusters to the control group (IO2, OM1, BM3, AT1, AA4, and KM3).

### 2.12. Allocation Concealment Mechanism

To ensure proper allocation concealment, a statistician will be assigned to produce the allocation sequence list by using block randomization software. Each cluster will be given a unique code in a sealed opaque envelope. Following that, the research assistant will open the envelopes and assign the clusters to the intervention or control group based on the list of codes generated by the software. Moreover, to avoid bias at the individual level, the envelopes will be opened only after the school coordinator selects the required participant number, and after obtaining consent for treatment and collecting baseline data.

### 2.13. Statistical Analyses

IBM SPSS Software 25.0 (SPSS Inc. Chicago, IL, USA) will be used to analyze collected data. However, missing data will be managed by adopting the intention-to-treat analysis concept. Prior to analysis, continuous variables will be checked for normality. The alpha level of significance will be set at the value of <0.05. Descriptive analysis will be employed to describe the data at baseline. Regarding the between-group comparisons, the chi-square test will be applied to compare the frequency difference of categorical data of the intervention with that of the control groups. The independent-samples *t*-test will be applied to compare the mean difference of continuous data of the intervention with that of the control group. 

Furthermore, to evaluate the changes within both groups, Cochran’s Q test will be applied to test the frequency differences of categorical data within the study groups’ overtimes. The one-way repeated measures ANOVA will be applied to identify the mean difference within the study groups for continuous variables overtimes. Generalized estimating equations will be used to test the main effect and interaction between and within the overtimes of the intervention and control groups.

### 2.14. Ethical Consideration

Ethical clearance was obtained from Jawatankuasa Etika Universiti Untuk Penyelidikan Melibatkan Manusia (JKEUPM) at Universiti Putra Malaysia [Ref No. FPSK(EXP16)P151] and the participating schools. This study will follow the ethical criteria throughout its entire procedure. Participants will be assured that their participation in the study is voluntary, and that they have the full right to withdraw from the study at any time they please. A participant consent form will be signed by each participant prior to conducting the survey. Data will be held in a confidential and anonymous manner. This study was registered at ACTRN: ACTRN12618000173291).

## 3. Discussion

This study presents a protocol aimed at examining the impact of BCS educational intervention on the BCS practice uptake, knowledge, and health beliefs of Yemeni female school teachers in Malaysia. It is believed that late-stage BC imposes extremely serious burdens on patients and their families. It further causes economic burdens on employers, communities, and governments. Our educational program in this regard serves as a plan in BC prevention through engaging an underserved population. The aim is to improve BCS outcomes and reduce disparities. Accordingly, this study intends to contribute to the credibility and effectiveness of utilizing a theory-based intervention aiming at educating women about BCS. The expected findings offer a better understanding of the role of knowledge and beliefs in promoting BCS uptake. Not only do the findings directly benefit teachers, their students, families, and friends, but also inform researchers and health care workers who strive to produce interventions serving women. The knowledge gained from this study could be used to plan appropriate BCS programs for other women in different contexts. These programs strengthen health systems with the intention to widely spread appropriate knowledge in communities. They could be utilized to ensure awareness of targeted groups at both the individual and social levels. To the best of the researchers’ knowledge, this study is the first of its kind that evaluates BCS educational intervention using a theoretical framework among Yemeni school teachers in Malaysia. If proven effective, this educational intervention will be evaluated and implemented in a diversity of settings that provide health education interventions for women. 

## 4. Implication for Practice

Though there is no proof that BSEs reduces BC mortality rates, it should be encouraged to detect BC in women efficiently. Moreover, although BSE may be an essential way to raise breast awareness, women are still asked to bear the responsibility for their own health by examining themselves. This is in order for them to be familiar with their breasts, observe and feel any changes from the norm, and consequently report any apparent changes immediately. Along with raising women’s awareness to undergo mammography, BSE may also involve the first stage in decreasing the barriers preventing women from undergoing CBE and mammography. In addition, CBE might create an opportunity to discuss screening matters with physicians. Thus, proper educational interventions are required to urge women to adhere to regular breast awareness and BCS examinations.

## 5. Conclusions

To recapitulate, this study examines the effects of BCS educational intervention on the BCS practice uptake, knowledge, and health beliefs of Yemeni female school teachers in Klang Valley, Malaysia using a cRCT design. The current study has a twofold aim. On the one hand, it aims to develop an educational intervention grounded in the HBM on BCS uptake, knowledge, and beliefs. On the other hand, it aims to assess the effectiveness of the educational intervention on BCS uptake, knowledge, and beliefs among Yemeni female school teachers in Klang Valley, Malaysia, in different time points (baseline, immediately, three- and six-months). In other words, since teachers play a vital role in educating and motivating young generations and future societies, it is highly important to provide them better health education.

## Figures and Tables

**Table 1:** Outline of the educational intervention on breast cancer screening.

Sessions	Topics	HBM Constructs	Area of Target	Intervention
The normal breast	- Structure of the breast - Breast development	- Perceived susceptibility	- Knowledge of breast cancer	- PowerPoint presentation - Booklet
Knowledge of breast cancer	- What is breast cancer - Symptoms of breast cancer - Breast cancer stages - Breast cancer risk factors	- Perceived susceptibility - Perceived seriousness	- Knowledge of breast cancer	- PowerPoint presentation - Booklet - Testimony film
Breast cancer screening	- Clinical breast examination - Mammography	- Perceived benefits - Perceived barriers - Health motivation (Cue to action)	- Knowledge and beliefs on breast cancer screening	- PowerPoint presentation - Testimony film - Booklet - BC logo sticker - Short reminder SMS
Breast health awareness	- Breast health awareness - BSE performance	- Perceived benefits - Perceived barriers - Health motivation (Cue to action) - Confidence	- Knowledge and beliefs on breast health awareness - Practice of BSE	- PowerPoint presentation - Testimony film - BSE film - Booklet - CD - BC logo sticker - Short reminder SMS
Practice	- Practice of BSE	- Perceived benefits - Perceived barriers - Health motivation (Cue to action) - Confidence	- Practice of BSE	- BSE practice on a silicon model - BSE film - Booklet - CD - BC logo sticker - Short reminder SMS

The educational intervention on BCS was instigated due to the high prevalence of BC. It is grounded in the theoretical framework of the HBM. Our priorities are to improve the perception of the six constructs of the HBM and to address knowledge regarding BC and BCS. The expected outcome is an increase in BC screening uptake due to improving knowledge and health beliefs. The educational intervention was introduced in the form of a PowerPoint presentation, a BSE training session, and distribution of a booklet, CD, and a breast cancer logo.

## Abbreviations

BC	Breast cancer
BCS	Breast cancer screening
BSE	Breast self-examination
CBE	Clinical breast exam
HBM	Health Belief Model
Crct	Cluster-randomized controlled trial
CVI	Content validity index
